# Serum Serotonin Levels as a Potential Risk Factor for Overactive Bladder in a Community‐Dwelling Population: A Four‐Year Longitudinal Study

**DOI:** 10.1111/luts.70019

**Published:** 2025-06-28

**Authors:** Takafumi Fukushima, Teppei Okamoto, Tomoko Hamaya, Hirotake Kodama, Naoki Fujita, Hayato Yamamoto, Atsushi Imai, Shigeyuki Nakaji, Shingo Hatakeyama

**Affiliations:** ^1^ Department of Urology Hirosaki University Graduate School of Medicine Hirosaki Japan; ^2^ Department of Social Medicine Hirosaki University Graduate School of Medicine Hirosaki Japan

**Keywords:** overactive bladder, risk factor, serum serotonin

## Abstract

**Purpose:**

This study aims to investigate the potential influence of serum serotonin (5‐HT) levels on the development of overactive bladder (OAB) in a community‐dwelling population.

**Methods:**

A four‐year longitudinal study was conducted involving 615 subjects who participated in the Iwaki Health Promotion Project in Hirosaki, Japan, in both 2015 and 2019. OAB was defined as experiencing urinary urgency at least once a week with an Overactive Bladder Symptom Score (OABSS) of ≥ 3. Baseline data from 2015, including serum 5‐HT levels, other laboratory data, and comorbidity information, were used for the analysis. The association between serum 5‐HT levels and incident OAB in 2019 (OAB‐2019) was examined using multivariate logistic regression analyses.

**Results:**

The study included 250 men and 365 women, of whom 74 individuals (29 men and 45 women) met the diagnostic criteria for OAB in 2019. Significant differences were observed between the OAB‐2019 and non‐OAB‐2019 groups, including age, chronic kidney disease, irritable bowel syndrome, hypertension, glycemic status, mental status, and OAB in 2015. Participants in the OAB‐2019 group had significantly lower serum 5‐HT levels compared to the non‐OAB‐2019 group (100 vs. 129 ng/mL, *p* < 0.001). After adjusting for confounders, multivariable analysis revealed that serum 5‐HT levels < 134 ng/mL (odds ratio: 2.48, 95% confidence interval: 1.39–5.83, *p* = 0.004), age, mental status, and OAB in 2015 independently served as risk factors for OAB‐2019.

**Conclusions:**

Low serum 5‐HT levels may be associated with an increased risk of OAB. Further research is needed to elucidate the underlying mechanisms responsible for this association.

## 1 Introduction

Overactive bladder (OAB) is a condition characterized by symptoms such as frequent urination, urgency, and nocturnal urination, which can occur with or without incontinence and significantly impact an individual's quality of life [1]. One prominent theory regarding its underlying mechanisms suggests that abnormal activation of the sensory pathways in the bladder leads to increased urinary urgency even when the bladder is not full [2].

Serotonin (5‐hydroxytryptamine [5‐HT]), primarily synthesized from L‐tryptophan in the gut and brainstem, plays a critical role as a neurotransmitter in the process of urination. The 5‐HT receptors are classified into seven families and are involved in both sensory and motor functions of the lower urinary tract, including the detrusor muscle and the external urethral sphincter, as well as the central pathways that control the micturition reflex [3, 4]. Central 5‐HT pathways are thought to have an inhibitory role in regulating urination, suggesting that changes in cerebrospinal fluid (CSF) 5‐HT levels may affect OAB [3]. Additionally, certain types of 5‐HT receptors are present in the urothelium and may contribute to bladder sensory perception, indicating a potential link between urinary 5‐HT levels and OAB pathophysiology [5, 6]. Our previous cross‐sectional study found that lower serum 5‐HT levels were independently associated with the presence of OAB [7]. However, the causal relationship between serum 5‐HT levels and OAB remains unclear. We hypothesized that lower serum 5‐HT levels might be a risk factor for developing OAB in the future. In this study, we longitudinally assessed the relationship between serum 5‐HT levels and future OAB development in a community‐dwelling population.

## 2 Materials and Methods

### 2.1 Design and Ethics Statement

This study adhered to the ethical standards outlined in the Declaration of Helsinki. Approval for using data from the Iwaki Health Promotion Project was granted by the Ethics Committee of Hirosaki University School of Medicine (authorization number 2018‐062). Since its inception in 2005, this annual health survey has been conducted for residents in the Iwaki area of Hirosaki city, targeting individuals aged 20 years and older who voluntarily participate. The primary objectives of the Iwaki project include preventing lifestyle‐related diseases, promoting health‐conscious lifestyles, and improving life expectancy among the residents of Hirosaki.

### 2.2 Data Collection and Evaluation of Variables

Participants provided written informed consent and answered questions about their lifestyle and personal information. They also underwent various tests, including blood sampling. Blood samples were collected in the morning, centrifuged immediately to obtain serum, and stored in EDTA‐containing tubes at −80°C until analysis. Serum 5‐HT levels (reference range 53–200 ng/mL) were measured using platelet‐rich plasma. Lipid profiles and hemoglobin A1c (HbA1c) were analyzed using standard laboratory methods.

In 2015, 1113 individuals participated in the study, with 656 continuing in 2019. Baseline data were extracted from the 2015 database. The study recorded medical histories of cardiovascular disease (CVD), hypertension, chronic kidney disease (CKD) (defined as an estimated glomerular filtration rate < 60 mL/min/1.73 m^2^), dyslipidemia, irritable bowel syndrome (IBS), and type 2 diabetes mellitus (DM). Sleep quality was assessed using the Pittsburgh Sleep Quality Index Japanese version, with sleep disturbance defined as a PSQI score exceeding five [8]. To assess oxidative stress markers, we examined urine creatinine‐adjusted urine 8‐Hydroxy‐2′‐deoxyguanosine (8‐OHdG) levels using the New 8‐OHdG Check ELISA kit. Morning urine samples were stored at −80°C until analysis. Visceral fat area was calculated using abdominal bioelectrical impedance analysis. Mental status was assessed using the Short‐Form 36‐Item Health Survey (SF‐36) Japanese version, a validated tool for measuring health‐related quality of life [9]. The SF‐36 is a scientifically validated and reliable instrument used to measure health‐related quality of life. Lower SF‐36 scores indicate poorer mental status. Participants lacking crucial data for analysis or taking serotonin selective reuptake inhibitors (SSRIs) and serotonin noradrenaline reuptake inhibitors (SNRIs) were excluded due to potential impacts on serum 5‐HT levels and micturition. The reason for this exclusion was that these medications can desensitize certain types of 5‐HT receptors in the brainstem, potentially leading to reduced serum 5‐HT levels and influencing micturition [10, 11].

### 2.3 Evaluation of OAB and Classification of Participants

OAB was assessed using the Overactive Bladder Symptom Score (OABSS) as presented in Table S1. The OABSS consists of four individual questions related to OAB symptoms, each measured on its own scale. Q1 assessed daytime frequency, Q2 evaluated nocturia, Q3 examined urinary urgency, and Q4 addressed urge incontinence. Scores range from 0 to 15, with higher scores indicating more severe OAB. OAB was defined as having two or more points in urinary urgency and an overall OABSS score of three or higher. Participants were categorized into OAB and non‐OAB groups based on these scores. All participants who were analyzed answered the OABSS questionnaire both in 2015 and 2019.

### 2.4 Statistical Analysis

Statistical analyses were performed using GraphPad Prism 5.03 and EzR: R commander version 1.6–3. Normally distributed continuous variables were presented as means with standard deviations (SD), while non‐normally distributed variables were expressed as medians with interquartile ranges (IQR). Categorical variables were compared using the χ^2^ test or Fisher's exact test. Differences between groups were evaluated using Student's *t*‐test for normally distributed data and Mann–Whitney U test for non‐normally distributed data. Multivariable logistic regression was used to analyze associations between factors and OAB‐2019.

## 3 Results

### 3.1 Baseline Characteristics

**FIGURE 1:**
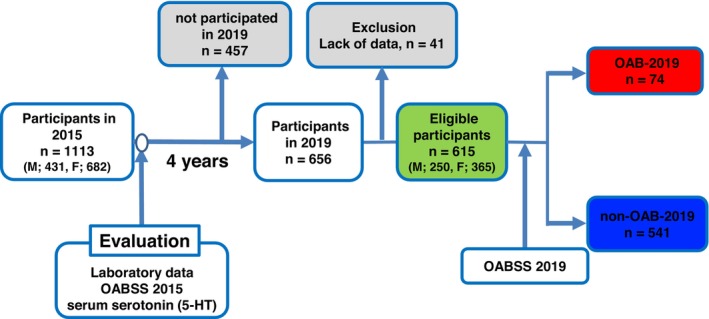
Analyzed participants in this study. Of the 1113 participants in the year of 2015, 656 participated in 2019. Of those participants, we excluded 41 who lacked crucial data for further analysis. Finally, we evaluated 615 (250 male and 365 female) individuals.

Out of the 1113 participants surveyed in 2015, 656 were reassessed in 2019. After excluding 41 individuals with incomplete data, a total of 615 participants (250 men and 365 women) were included in the final analysis. The median serum serotonin (5‐HT) concentration was 124 ng/mL (IQR: 93–159 ng/mL). Among the participants, 74 (29 men and 45 women) were identified as having OAB in 2019, while the remaining 541 were categorized as non‐OAB (Figure 1).

### 3.2 Comparison of Characteristics Between the OAB and Non‐OAB Groups

**FIGURE 2:**
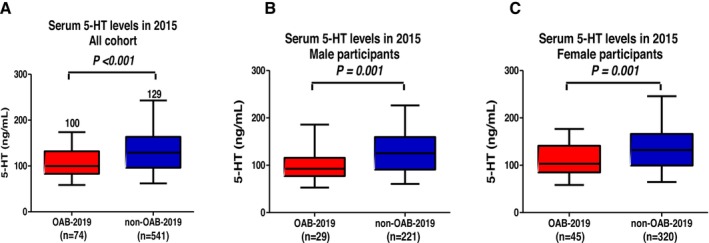
Comparison of serum serotonin (5‐hydroxytryptamine (5‐HT)) levels between overactive bladder (OAB)‐2019 and non‐OAB‐2019. All cohort (A), male participants (B), female participants (C). The participants in the OAB‐2019 group demonstrated significantly lower serum 5‐HT levels in comparison to the non‐OAB‐2019 group.

Table 1 outlines the baseline characteristics of the study participants, separated into OAB and non‐OAB groups for 2019. The OAB group was significantly older (*p* < 0.001), had higher HbA1c levels (*p* = 0.003), and reported lower mental health scores on the SF‐36 scale (*p* = 0.007) compared to the non‐OAB group. Additionally, the OAB group had a higher prevalence of CKD, IBS, hypertension, and a history of OAB from 2015. Serum 5‐HT levels were markedly lower in the OAB group compared to the non‐OAB group (100 vs. 129 ng/mL, *p* < 0.001; Figure 2A). In male participants, the OAB‐2019 group showed lower serum 5‐HT levels than the non‐OAB‐2019 group (93 vs. 125 ng/mL, *p* = 0.001; Figure 2B). Similarly, in female participants, the OAB‐2019 group exhibited lower serum 5‐HT levels compared to the non‐OAB‐2019 group (103 vs. 132 ng/mL, *p* = 0.001; Figure 2C).

**TABLE 1:** Comparison of baseline characteristics between the OAB‐2019 and non‐OAB‐2019 group.

	OAB‐2019	Non‐OAB‐2019	*p*
Number of participants	74	541	—
Male sex^a^ (presence), *n* (%)	29 (40%)	221 (41%)	0.802
Age^b^ (years) (median, IQR)	65 (58–71)	53 (41–62)	< 0.001
History of CVD^a^ (presence), *n* (%)	7 (9.5%)	30 (5.5%)	0.191
Hypertension^a^ (presence), *n* (%)	33 (45%)	158 (29%)	0.010
DM^a^ (presence), *n* (%)	8 (11%)	35 (6.5%)	0.219
CKD^a^ (presence), *n* (%)	21 (28%)	67 (12%)	0.001
Dyslipidemia^a^ (presence), *n* (%)	29 (39%)	214 (40)	1.000
IBS^a^ (presence), *n* (%)	11 (15%)	39 (7.2%)	0.038
PSQI > 5^a^ (presence), *n* (%)	19 (26%)	105 (19%)	0.217
Smoking history^a^ (presence), *n* (%)	25 (34%)	213 (39%)	0.376
Habitual drinking history^a^ (presence), *n* (%)	34 (46%)	245 (45%)	1.000
BMI^b^ (kg/m^2^) (median, IQR)	23.0 (21.0–24.4)	22.3 (20.1–24.6)	0.351
OAB in 2015^a^ (presence), *n* (%)	42 (57%)	32 (5.7%)	0.001
HbA1c^b^ (%) (median, IQR)	5.8 (5.5–6.1)	5.6 (5.4–5.9)	0.003
SF‐36 mental health^b^ (ng/mL) (median, IQR)	50.4 (41.8–57.1)	51.8 (46.5–59.8)	0.007
Visceral fat area (cm^2^)	82 (53–114)	76 (50–105)	0.461
Urine 8‐OHdG	5.6 (4.4–7.3)	5.2 (3.8–6.6)	0.050
LDL‐Chol^b^ (%) (median, IQR)	116 (98–133)	117 (97–135)	0.810

Abbreviations: 8‐OHdG, 8‐Hydroxy‐2′‐deoxyguanosine; BMI, body mass index; CKD, chronic kidney disease; CVD, cardiovascular disease; DM, diabetic mellitus; eGFR, estimated glomerular filtration rate; HbA1c, hemoglobin A1c; IBS, irritable bowel syndrome; IQR, interquartile range; LDL, low‐density lipoprotein; OAB, overactive bladder; PSQI, Pittsburgh sleep quality index.

^a^ *χ* ^2^ test.

^b^ Mann–Whitney *U* test.

### 3.3 Subgroup Analysis

**FIGURE 3:**
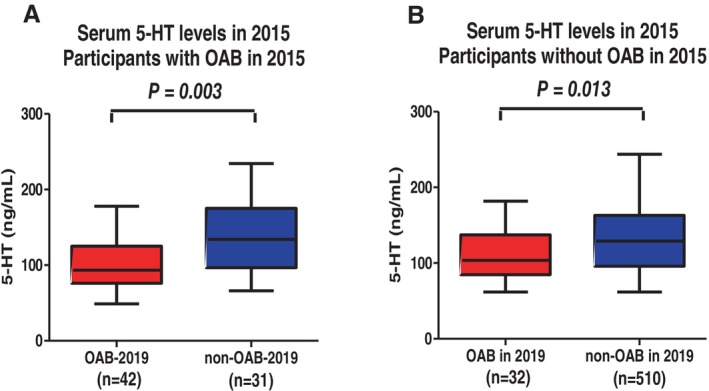
Comparison of serum serotonin (5‐hydroxytryptamine (5‐HT)) levels between overactive bladder (OAB)‐2019 and non‐OAB‐2019, according to OAB status in 2015. Participants who met the diagnostic criteria for OAB in 2015 (A). Those who continued to have OAB symptoms in 2019 had significantly lower serum 5‐HT levels compared to the non‐OAB‐2019. Participants who met the diagnostic criteria for OAB in 2015 (B). Those who newly met the criteria for OAB in 2019 had significantly lower serum 5‐HT levels compared to the control group.

In participants who met the criteria for OAB in 2015, those who continued to exhibit OAB symptoms in 2019 (OAB‐2019 group) had significantly lower serum 5‐HT levels than those whose symptoms had resolved (94 vs. 134 ng/mL, *p* = 0.003; Figure 3A). Similarly, among participants who did not meet the criteria for OAB in 2015, those who developed OAB by 2019 had lower serum 5‐HT levels compared to the control group (103 vs. 129 ng/mL, *p* = 0.013; Figure 3B).

### 3.4 Multivariable Logistic Regression Analyses for an OAB‐2019

Multivariable logistic regression analysis identified serum 5‐HT levels, age, mental health scores from SF‐36, and OAB status in 2015 as independent factors associated with OAB‐2019 (Table 2, Model 1). The optimal threshold for serum 5‐HT levels to predict OAB‐2019 was determined to be 134 ng/mL, yielding a sensitivity of 0.63 and specificity of 0.62 (Figure S1). Serum 5‐HT levels below this cutoff were associated with a 2.83‐fold higher risk of developing OAB in 2019 (OR: 2.83, 95% CI: 1.39–5.83, *p* = 0.004; Table 2, Model 2).

**TABLE 2:** Multivariable logistic analysis of OAB‐2019.

Model 1				
Variable	Risk factor	*p*	OR	95% CI
Age (years)	Continuous	< 0.001	1.06	1.03–1.10
Male sex	Presence	0.740	1.12	0.59–2.13
BMI (kg/m^2^)	Continuous	0.680	0.98	0.88–1.08
IBS	Presence	0.090	2.33	0.88–6.19
CKD	Presence	0.280	1.54	0.70–3.40
HbA1c (%)	Continuous	0.990	1.00	0.57–1.75
SF‐36 mental health	Continuous	0.031	0.96	0.93–0.99
OAB in 2015	Presence	< 0.001	15.2	7.95–29.2
Hypertension	Presence	0.220	0.64	0.31–1.31
Serum 5‐HT (ng/mL)	Continuous	0.002	0.99	0.98–1.00
**Model 2**				
Serum 5‐HT (ng/mL)	< 134 ng/mL	0.004	2.83	1.39–5.83

Abbreviations: BMI, body mass index; CKD, chronic kidney disease; HbA1c, hemoglobin A1c; HT, 5‐hydroxytryptamine; IBS, irritable bowel syndrome; OAB, overactive bladder.

## 4 Discussion

This study aimed to evaluate the potential influence of serum serotonin (5‐HT) levels on the risk of developing OAB in a community‐based population. Our findings revealed that individuals who developed OAB over the four‐year study period (OAB‐2019 group) had significantly lower serum 5‐HT levels compared to those who did not. Multivariable logistic regression analysis further confirmed that reduced serum 5‐HT levels were independently associated with an elevated risk of OAB. These results suggest that diminished serum 5‐HT levels may play a role in the pathogenesis of OAB.

The serotonergic system plays a complex role in regulating micturition. Serotonergic neurons originating in the brainstem's raphe nuclei project to various areas of the brain and spinal cord, modulating bladder control via multiple 5‐HT receptor subtypes. For instance, stimulation of the raphe nuclei inhibits bladder activity by acting on 5‐HT receptors in the lumbosacral spinal cord [3]. Stimulation of the raphe nuclei inhibits micturition by acting on 5‐HT receptors found on parasympathetic neurons in the lumbosacral cord [3]. In the spinal cord, specific 5‐HT receptor subtypes, namely 5‐HT1A and 5‐HT7, have excitatory effects on micturition, while 5‐HT2A and 5‐HT2C receptors have inhibitory effects [12, 13, 14]. Additionally, the bladder's peripheral nervous system expresses various 5‐HT receptors, playing a role in regulating detrusor contraction [4]. Some animal experiments have suggested that 5‐HT can directly induce the contraction of ureteral strips [15], indicating the involvement of postjunctional 5‐HT receptors in facilitating detrusor contraction. Conversely, the stimulation of 5‐HT1D receptors in the urothelium was found to inhibit the release of adenosine triphosphate, which is associated with bladder hyperactivity, in a rat model [5]. Furthermore, an animal experiment in mice revealed that 5‐HT4 receptor expressed on uroepithelial cells plays an inhibitory role in mechanosensory transduction in the bladder. The loss of 5‐HT4 receptor‐mediated inhibition may enhance bladder afferent sensitivity and exacerbate bladder overactivity [16]. These results may support the idea that certain types of 5‐HT receptors can suppress bladder hyperactivity. However, the precise roles of 5‐HT receptors and the interactions between different receptor subtypes in the micturition reflex remain incompletely understood. Further research is required to fully grasp the implications of our findings concerning micturition control within the central serotonergic system.

The clinical significance of serum 5‐HT levels in lower urinary tract symptoms (LUTS) should be discussed further. Consistent with our previous study [7], several research works have established a link between lower 5‐HT levels in serum and CSF and LUTS [17, 18]. It is known that CSF 5‐HT levels are positively correlated with its levels in serum and urine [19]. Hence, it is reasonable to speculate that reduced serum 5‐HT levels may reflect decreased 5‐HT activity in CSF and urine. However, these clinical basic researches could not determine the cause and effect association between 5‐HT levels and LUTS. Our current study revealed that participants in the OAB‐2019 group exhibited significantly lower serum 5‐HT levels compared to those in the non‐OAB‐2019 group. Furthermore, our study implied that participants with serum 5‐HT levels < 134 ng/mL had a 2.83‐fold increased risk of developing OAB after a 4‐year period. As illustrated in Figure 3A, individuals who experienced persistent OAB symptoms over the 4‐year period demonstrated lower serum 5‐HT levels compared to those whose OAB symptoms resolved. In addition, individuals who developed new OAB symptoms after 4 years exhibited lower serum 5‐HT levels compared to those who did not develop OAB (Figure 3B). These findings suggest that serum 5‐HT levels could serve as an indicator of OAB symptom remission and newly onset of OAB.

Interestingly, although the serum 5‐HT levels in participants who later developed OAB were significantly lower than those in the non‐OAB group, these values still fell within the standard reference range (53–200 ng/mL). This raises the question of the clinical relevance of “low‐normal” serotonin levels. We speculate that the pathological threshold of serum 5‐HT may differ depending on the target organ system or disease state. In the context of bladder function, even a modest reduction in serotonergic activity—while still within the general reference range—may be sufficient to disturb the inhibitory regulation of micturition and predispose individuals to OAB. Therefore, relatively low‐normal serum 5‐HT levels might still carry physiological significance specific to OAB pathophysiology. Further research is warranted to explore whether disease‐specific reference thresholds for serum serotonin should be considered in this context.

The factors associated with increases or decreases in serum 5‐HT levels should be investigated. A previous study showed that serum 5‐HT levels were negatively correlated with fat mass and age [20]. We also observed independent negative correlations with age and visceral fat area in a multiple regression analysis of serum 5‐HT levels (Figure S1B). Although the present longitudinal study suggests an association between lower serum 5‐HT levels and the future onset of OAB, it does not definitively establish whether low serotonin levels are a causal factor or merely a secondary consequence of OAB or its associated conditions, such as mental health disorders or metabolic dysfunction. However, in our previous cross‐sectional study involving a similar population [7], we found that serum 5‐HT levels were independently associated with the presence of OAB, even after adjusting for depressive symptoms using the CES‐D score, which showed no significant relationship with serum 5‐HT levels. These findings suggest that the relationship between serotonin and OAB is likely independent of mental health status. Although the precise mechanisms remain unclear, our current and previous findings consistently indicate a potential role of serotonin in the pathophysiology of OAB. We excluded nine participants from the study who were taking SSRIs or SNRIs since these medications can elevate 5‐HT levels in the CSF, potentially altering serum 5‐HT levels. In fact, the median value of serum 5‐HT levels for this subgroup was 12.2 ng/mL (data not shown), which significantly differed from the participants who were not on these medications (124 ng/mL). These findings contradict the previously reported positive correlation between CSF and serum 5‐HT levels among healthy volunteers. Several observational studies have indicated a possible association between the use of SSRIs and/or SNRIs and worsened LUTS [11, 21]. However, it is worth noting that duloxetine, a type of SNRI, has been reported to improve urge urinary incontinence [22, 23]. Further research is needed to elucidate the factors that influence systemic 5‐HT levels and their effect on LUTS.

This study has several limitations. The underlying mechanism responsible for the reduction in serum 5‐HT levels among participants with OAB remains unknown. Further research is needed to determine whether increasing serum 5‐HT levels improves OAB symptoms. One important limitation of our study is the lack of follow‐up data on serum 5‐HT levels in 2019. This prevented us from evaluating temporal changes in serotonin levels and whether such changes may be influenced by the development or progression of OAB. Future longitudinal studies with repeated measurements of serum 5‐HT are needed to clarify the causal and temporal relationships. There is a lack of information regarding the number of participants who were actually scrutinized and diagnosed with OAB. Important aspects such as dietary information, which could be deeply associated with OAB and 5‐HT levels, were not obtained in this study. Regional bias may have influenced the findings. The study's participants might predominantly consist of individuals with milder comorbidities who were already health‐conscious. In conclusion, the low serum 5‐HT levels may be linked to an increased risk of developing OAB. Further study is warranted to clarify an exact relationship between OAB and serum 5‐HT levels.

## Author Contributions

Takafumi Fukushima contributed to writing – review and editing, validation, resources, investigation, and data curation. Teppei Okamoto was involved in writing – review and editing, writing – original draft, validation, resources, investigation, data curation, and conceptualization. Tomoko Hamaya, Hirotake Kodama, Naoki Fujita, Hayato Yamamoto, and Atsushi Imai contributed to writing – review and editing. Shingo Hatakeyama contributed to writing – review and editing as well as supervision. Shigeyuki Nakaji provided supervision. All the authors read and approved the final manuscript.

## Disclosure

The authors have nothing to report.

## Ethics Statement

This study was conducted in accordance with the Declaration of Helsinki and approved by the Ethics Committee of Hirosaki University School of Medicine (approval number: 2018‐062). All participants provided written informed consent prior to their inclusion in the study.

## Consent

Informed consent was obtained from all subjects involved in the study. Written informed consent was obtained from the participants to publish this paper.

## Conflicts of Interest

The authors declare no conflicts of interest.

## Supporting information


**FIGURE S1.** The receiver operating characteristic curve of serum 5‐HT levels for OAB‐2019 (A). This analysis showed the area under curve was 0.663 (95% CI; 0.603–0.723).
**TABLE S1.** The multiple regression analysis of serum 5‐HT levels (B). Independent negative correlations with age and visceral fat area.

## Data Availability

Due to privacy and ethical restrictions, certain data may not be publicly accessible.
