# Supplementary material for: Serum Serotonin Levels as a Potential Risk Factor for Overactive Bladder in a Community‐Dwelling Population: A Four‐Year Longitudinal Study
Source: Low Urin Tract Symptoms. 2025 Jun 28;17(4):e70019. doi: 10.1111/luts.70019 (PMC12205573; doi:10.1111/luts.70019)
Supplement: Supplementary file 1 — FIGURE S1. The receiver operating characteristic curve of serum 5‐HT levels for OAB‐2019 (A). This analysis showed the area under curve was 0.663 (95% CI; 0.603–0.723). TABLE S1. The multiple regression analysis of serum 5‐HT levels (B). Independent negative correlations with age and visceral fat area. [file LUTS-17-e70019-s001.pptx]

## Slide 1
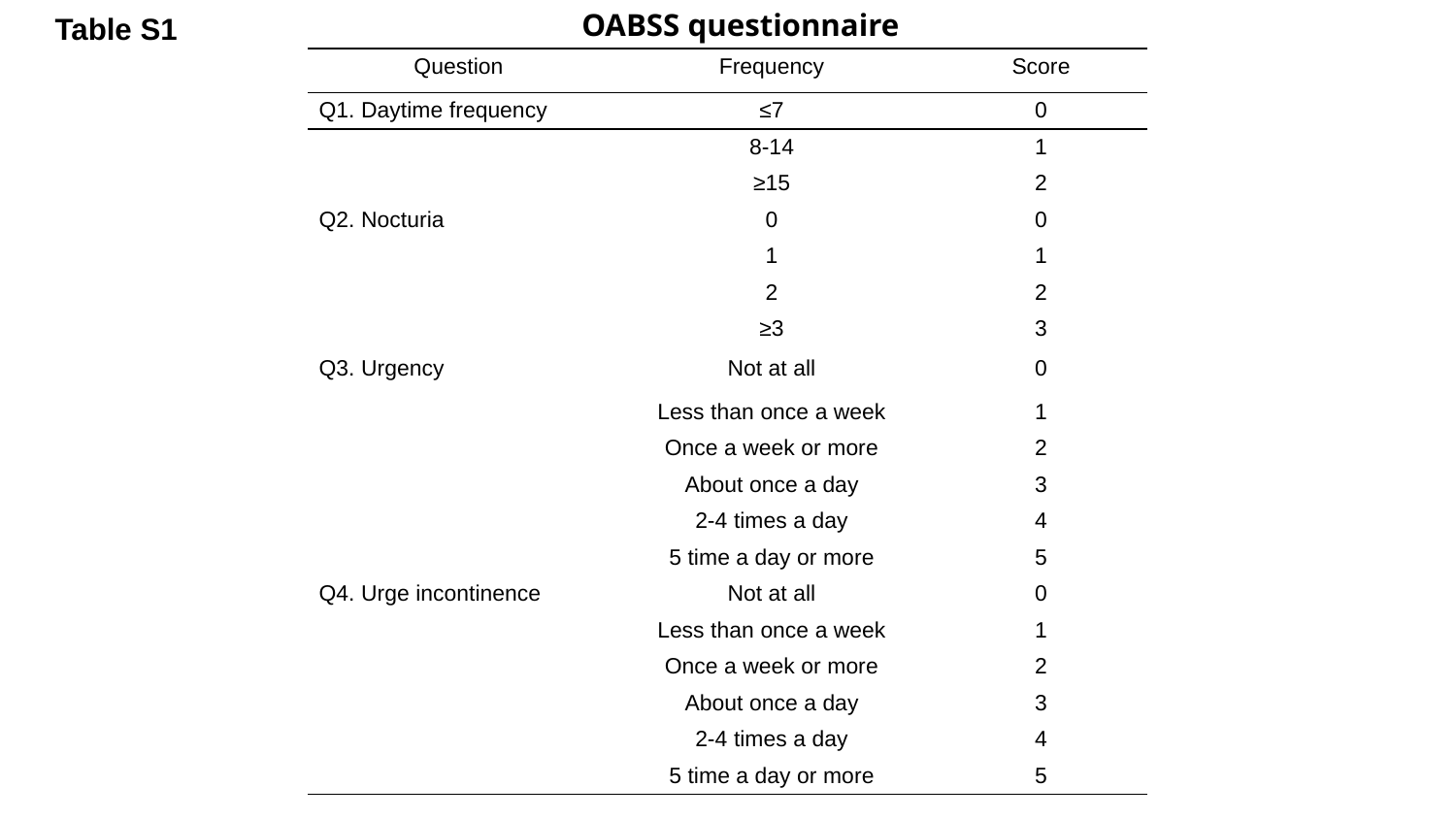

OABSS questionnaire
Table S1
| Question | Frequency | Score |
| --- | --- | --- |
| Q1. Daytime frequency | ≤7 | 0 |
| | 8-14 | 1 |
| | ≥15 | 2 |
| Q2. Nocturia | 0 | 0 |
| | 1 | 1 |
| | 2 | 2 |
| | ≥3 | 3 |
| Q3. Urgency | Not at all | 0 |
| | Less than once a week | 1 |
| | Once a week or more | 2 |
| | About once a day | 3 |
| | 2-4 times a day | 4 |
| | 5 time a day or more | 5 |
| Q4. Urge incontinence | Not at all | 0 |
| | Less than once a week | 1 |
| | Once a week or more | 2 |
| | About once a day | 3 |
| | 2-4 times a day | 4 |
| | 5 time a day or more | 5 |

## Slide 2
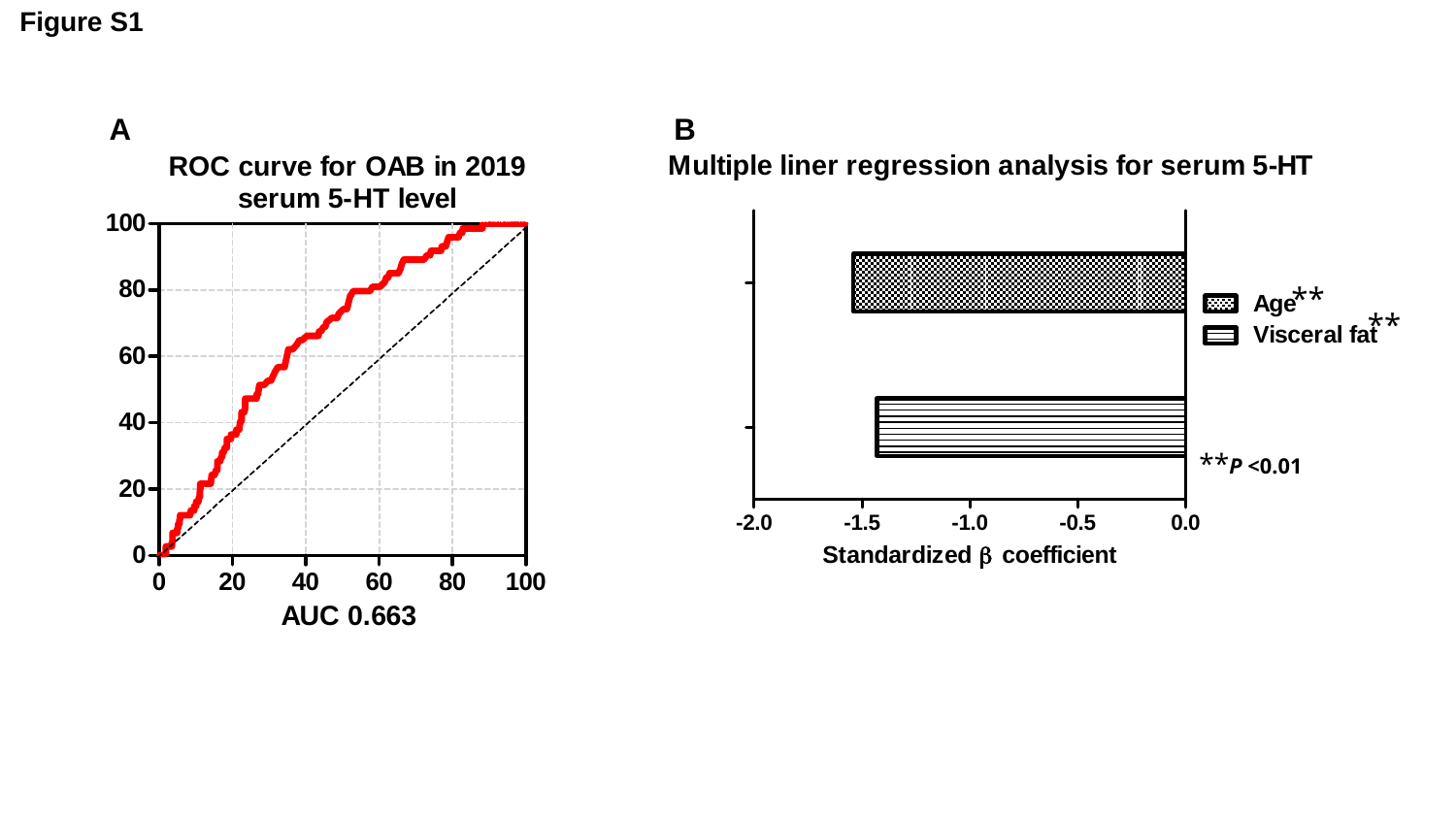

Figure S1
B
A
**
**
**P <0.01
